# Cytochrome P450 1B1: role in health and disease and effect of nutrition on its expression

**DOI:** 10.1039/c9ra03674a

**Published:** 2019-07-04

**Authors:** Bakht Ramin Shah, Wei Xu, Jan Mraz

**Affiliations:** University of South Bohemia in Ceske Budejovice, Faculty of Fisheries and Protection of Waters, South Bohemian Research Center of Aquaculture and Biodiversity of Hydrocenoses, Institute of Aquaculture and Protection of Waters Na Sádkách 1780 370 05 České Budějovice Czech Republic bshah@frov.jcu.cz +420 775022640; College of Life Science, Xinyang Normal University Xinyang 464000 People's Republic of China

## Abstract

This review summarizes the available literature stating CYP1B1 to provide the readers with a comprehensive understanding of its role in different diseases, as well as the importance of nutrition in their control in terms of the influence of different nutrients on its expression. CYP1B1, a member of the cytochrome P450 enzyme family is expressed in different human tissues and is known to contribute to different life alarming pathologies. Particularly, till now much attention has been paid to its involvement in the development of primary congenital glaucoma (PCG) and cancer. However, recently there are some reports highlighting CYP1B1 as a potential regulator in energy homeostasis and adipogenesis thus promoting obesity and hypertension as well. Therefore, seeking out effective strategies to modulate the expression of CYP1B1 is a challenging task. In this context, nutrients based strategies will be the best choice as they are mostly harmless and are easily available in one's diet. In conclusion, this article will be helpful in providing a base for further research that is needed to identify the role of CYP1B1 in progression of different diseases, hypertension and obesity in particular, and then to present the effectiveness, mechanisms, and biologic plausibility of nutrients against its expression.

## Abstract 2

This review summarizes the available literature stating CYP1B1 to provide the readers with a comprehensive understanding of its role in different diseases, as well as the importance of nutrition in their control in terms of the influence of different nutrients on its expression.

## Graphical abstract

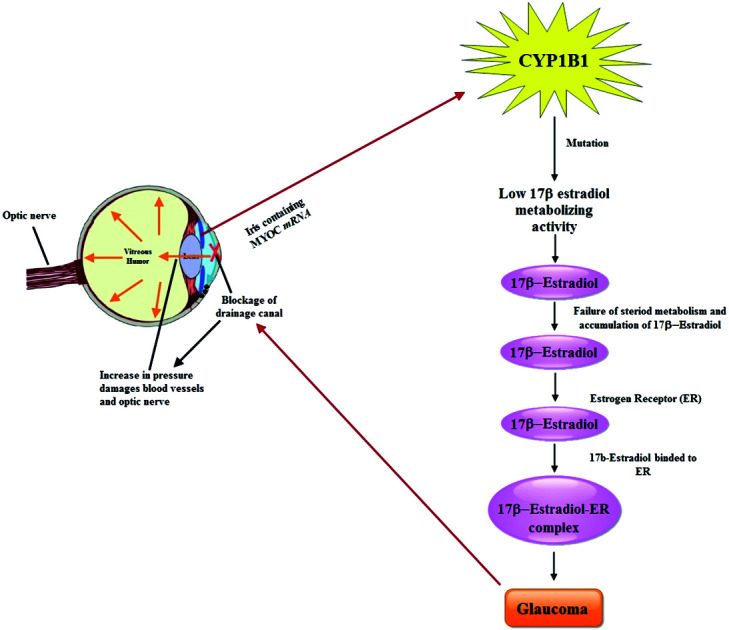

## Background

Cytochrome P450 enzymes are a large ubiquitous superfamily of enzymes, playing a significant physiological role in the detoxification of xenobiotics, and the biosynthesis of many endogenous compounds. Extensive research about “P-450” started in the late 1950s and 1960s, when P-450 was thought to be a single cytochrome, exclusively existing in liver,^1^ to metabolize drugs and other foreign chemicals.^2^ Recently it has been known that there are 18 mammalian cytochrome P450 (CYP) families, which encode 57 genes in the human genome. Among these, CYP2, CYP3 and CYP4 families contain far more genes than the other 15 families; these three families are also the ones that are dramatically larger in rodent genomes. Most (if not all) genes in the CYP1, CYP2, CYP3 and CYP4 families encode enzymes involved in eicosanoid metabolism and are inducible by various environmental stimuli (*i.e.* diet, chemical inducers, drugs, pheromones, *etc.*), whereas the other 14 gene families often have only a single member, and are rarely if ever inducible or redundant.^3^ However, among these, CYP 1B1 is one of the well-studied monooxygenases in humans and mammals.^4^

CYP1B1 is a member of the cytochrome P450 enzyme family 1, subfamily B and polypeptide 1. The story about CYP1B1 began back in 1994, when Sutter and co-workers isolated and characterized complete 5.1 kb cDNA corresponding to a 2,3,7,8-tetrachlorodibenzo-*p*-dioxin (TCDD)-responsive cDNA clone from a human keratinocyte cell line.^5^ Later on, Tang and coworkers differentiated CYP1B1 from the two others most closely related member of CYP 1 family *i.e.* CYP1A1 and CYP1A2, and determined its genomic structure.^6^

Previous studies reveal that CYP1B1 is expressed in many normal human tissues^5^*i.e.* liver,^7^ bone marrow,^8^ kidney,^9^ endocrine and reproductive organs^10^ and other tissues of various species,^11^ as well as in human blood monocytes and macrophages.^12^ However, its expression is conserved in the early embryo across several species during the development of the neural crest, hindbrain, and eyes.^13,14^

CYP1B1 plays an important role in the oxidative metabolism of xenobiotics, particularly bioactivation of polycyclic aromatic hydrocarbons. It metabolizes substrates of endogenous origin including retinol metabolism to retinal, the hydroxylation of melatonin, dietary plant flavonoids, and the formation of genotoxic catechol estrogens.^8,14^ Stoilov *et al.*,^15^ identified CYP1B1 as the gene affected in primary congenital glaucoma and presented to be the first example in which mutations in a member of the cytochrome P450 super family results in a primary developmental defect. Whereas, Murray and coworkers appear to be the first to demonstrate that CYP1B1 protein is present and overexpressed in human tumor tissues.^16^ It is believed that CYP1B1 primarily acts as a hydroxylase for 17β-estradiol at positions C2 and C4, and the products from these enzymatic reactions participate in metabolic processes that generate quinone metabolites involved in the production of carcinogenic DNA adducts.^17^ Recently, it has been determined that CYP1B1 enhances cell proliferation by inducing cell cycle transition and inhibiting cellular apoptosis in endometrial and breast cancer cells.^18^ Similarly, CYP1B1 expression was found to increase in the early phase of *in vitro* adipogenic differentiation, in parallel with PPARγ expression.^19^ The association of CYP1B1 activity with adipogenic PPARγ expression and decreased fatty acid peroxidation suggests that CYP1B1 may play an important role in energy homeostasis.^20^

The preceding discussion revealed that CYP1B1 can be the target in treatment and prevention of different pathologies. Many herbs contain numerous phytochemical constituents which can induce or inhibit drug metabolizing enzymes such as cytochrome P450 (CYP) enzymes. Particularly, these phytochemicals function as pro drugs for CYP1B1.^21^ However, unfortunately, CYP1B1 is rarely considered in discussions of the bioactivity of plant-based agents that affect cancer, which is a potentially important factor.^22^

Therefore, the present review was aimed to summarize the available literature describing the contribution of CYP1B1 in health and disease and the influence of nutrition on its expression.

## Role of CYP1B1 in glaucoma

Glaucoma, a group of eye diseases causing irreversible vision loss is the second leading cause of blindness, affecting ∼65 million people across the globe.^23^ Generally, the disease is classified into open-angle glaucoma, closed-angle glaucoma and primary congenital glaucoma (PCG) on the basis of age of onset, anatomy of the anterior chamber and etiology.^24^ PCG occurs in the neonatal or early infantile stages and is characterized by symptoms like enlargement of the globe, opacification of the cornea, and breaks in Descemet's membrane,^25^ and causes non-recoverable blindness due to the loss of retinal ganglion cells. The expression of CYP1B1 plays an important role in the modulation of development and functions of the trabecular meshwork (TM). And mutation in this gene (CYP1B1) are implicated in the development of PCG in humans.^26^ The phenomenon was further explained by the same research group and Teixeira *et al.*, describing a significantly decreased amount of TM collagen and enhanced TM endothelial cell and collagen lesion scores in CYP1B1 knockdown (Cyp1b1^−/−^) mice than in age matched counterparts. The role of CYP1B1 mutations in the TM progression and PCG suggested a link between TM morphologic alterations and elevated intraocular pressure. Furthermore, Cyp1b1^−/−^ mice were found to have lower expression of periostin, elevated lipid peroxidation and abnormalities in their TM tissue having increased ROS.^27,28^ Also, deletion of constitutively expressed CYP1B1 attenuated retinal endothelial cell (EC) capillary morphogenesis (CM) *in vitro* and angiogenesis *in vivo* largely due to increased intracellular oxidative stress and increased production of thrombospondin-2 (TSP2), an endogenous inhibitor of angiogenesis. This linked CYP1B1 metabolism in retinal ECs with sustained endothelium nitric oxide synthase (eNOS) activity and nitric oxide (NO) synthesis and/or bioavailability and low oxidative stress and thrombospondin-2 expression.^29^ Whats more, CYP1B1 deficiency in EC and pericytes (PC) caused in enhancing oxidative stress, alterations in migration, attenuation of capillary morphogenesis, sustained activation of NF-κB, and enhanced expression of (TSP2). This vital role of TSP2 in modulation of NF-κB activity and attenuation of angiogenesis supported the claims of CYP1B1 expression in vascular cells involved in the regulation of vascular homeostasis through modulation of the cellular reductive stat, TSP 2 expression and NF-κB activation.^30^ Similarly, by evaluating the impact of CYP1B1 on retinal AC (astrocytes) function and regulating redox homeostasis, another research group showed that Cyp1b1^−/−^ retinal ACs were more proliferative and migratory. Hence CYP1B1 expression in ACs performs a crucial part in retinal neurovascular homeostasis and also in modulation of tissues integrity and function *via* regulation of cellular redox homeostasis and extracellular microenvironment.^31,32^

**Fig. 1:**
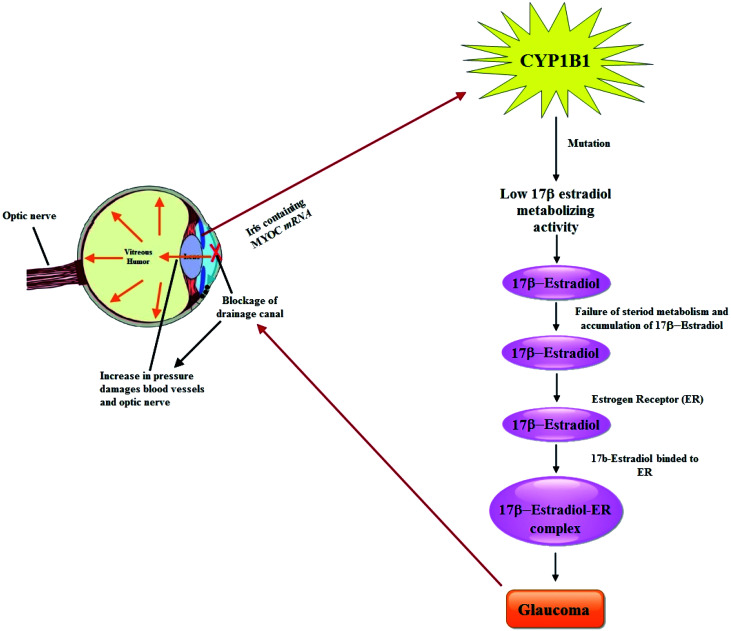
Schematic representation of the pathway in the pathology of glaucoma by CYP1B1.

It is postulated that PCG accounts for 55% of primary pediatric glaucoma and is an autosomal recessive disorder which is caused predominantly by mutations in the CYP1B1 gene.^33^ Until now, more than 150 mutations in CYP1B1 have been determined linked with PCG that account a significant fraction of the genetic load of familial and sporadic cases of PCG.^34^ Previous studies have shown that there are two chromosomal locations for PCG on 2p21 (GLC3A, MIM 231300) and 1p36 (GLC3B), where GLC3A locus colocalizes to the CYP1B1 gene (MIM 601771 or MYOC) on chromosome 2p21.^35^ Basically MYOC encodes a 504-amino-acid glycoprotein that contains an olfactomedin domain (residues 246–501) and is known to be the site where the majority (42/46 [91.3%]) of the mutations documented have been identified. Normally, in eyes, *MYOC* mRNA is expressed in the iris, ciliary body, and trabecular meshwork,^36^ as well as in retinal photoreceptor cells^37^ and optic nerve head—specifically, the astrocytes.^38^ Perfusing the trabecular meshwork with mutant recombinant protein results in an increase in outflow resistance.^39^ Huang *et al.*, investigated mutations in MYOC, OPTN, NTF4, WDR36 and CYP1B1 in a cohort of 67 unrelated Chinese Juvenile onset open-angle glaucoma (JOAG) patients. Among them, 2 heterozygous mutations in MYOC (c. 1109C > T, p. (P370L); c. 1150G > C, p. (D384H)), 2 heterozygous mutations in OPTN (c. 985A > G, p. (R329G); c. 1481T > G, p. (L494W)) and 2 homozygous mutations in CYP1B1 (c. 1412T > G, p. (I471S); c. 1169G > A, p. (R390H)) were identified as potentially causative mutations.^40^ A facile pathway of CYP1B1 involvement in the pathology of glaucoma has been drawn in Fig. 1.

Prevalence of PCG varies across different ethnic groups as estimated in early years to occur from 1 in 10 000–20 000 in the western populations,^41^ to 1 in 2500 and 1 in 1250 in the Saudi Arabian population^15^ and Gypsy population of Slovakia,^41^ respectively. And hence the researchers around the world have paid much attention to the disease in the late years.

In this context, Bassem *et al.*, (1998) determined three distinctive mutations (G → A transition at nucleotide 3987, C → T transition at nucleotide 8242, and G → A transition at nucleotide 7957) for cytochrome P4501B1 (*CYP1B1*) in 25 Saudi PCG families that were found to segregate with the phenotype in 24 families.^42^ Later on, Sitorus and research group^43^ analyzed CYP1B1 gene mutations in patients with PCG (GLC3A, OMIM 231300) in 21 Indonesian and European (white) patients (12 Indonesian, nine European). Amongst the Indonesian, 25% of the cases were found to be positive for theV364M mutation and interestingly a novel missense mutation (S215I) in exon 2 was also found in one of these patients. Similarly, a novel 12 bp deletion at nucleotide 1407 in exon 3 was characterized in a patient of Turkish descent. The 13 bp deletion at nucleotide 1410 was found in a patient of Italian descent. Their results concluded that the CYP1B1 gene is a major gene for PCG al (GLC3A) although a different pattern of CYPB1 mutations and benign sequence variants were found in Indonesian patients compared with European (white) patients.^43^ Similarly, Mukesh and coworkers evaluated the mutation profile of CYP1B1 in 50 North Indian primary congenital glaucoma (PCG) patients. They isolated genomic DNA from the blood and trabecular meshwork, and examined *CYP1B1* for the six predominant mutations (termination at 223 [Ter@223], Gly61Glu, Pro193Leu, Glu229Lys, Arg368His, andArg390Cys). Their results showed that 42% of the cases were positive for these mutations, whereas 46% harbored the *CYPIB1* mutations. Amongst these cases, 18%, 16% and 8% were found positive for mutations like Ter@223, p.R390H and p.R368H respectively.^33^

Recently, another research group investigated the contribution of CYP1B1 to the pathogenesis of PCG in the Pakistani population. They identified a total of 11 mutations including 7 missense mutations: p.Y81N, p.E229K, p.R368H, p.R390H, p.W434R, p.R444Q and p.R469W, as well as 1 nonsense mutation, p.Q37*, and 3 frameshift mutations, p.W246Lfs81*, p.T404Sfs30* and p.P442Qfs15*, reconfirming the genetic heterogeneity of CYP1B1 in the pathogenesis of PCG. They stated that all of these mutations segregated with the disease phenotype in their respective families.^34^ Researchers from Germany investigated 12 rare heterozygous missense mutations in TEK by targeted sequencing. Among them four of these TEK mutations (p.E103D, p.I148T, p.Q214P, and p.G743A) co-occurred with three heterozygous mutations in another major PCG gene CYP1B1 (p.A115P, p.E229K, and p.R368H) in five families. These findings let them suggest the interaction of TEK and CYP1B1 contributing to PCG pathogenesis and that TEK-CYP1B1 may perform overlapping as well as distinct functions in manifesting the disease etiology.^44^

## CYP1B1 a universal tumor marker

**Fig. 2:**
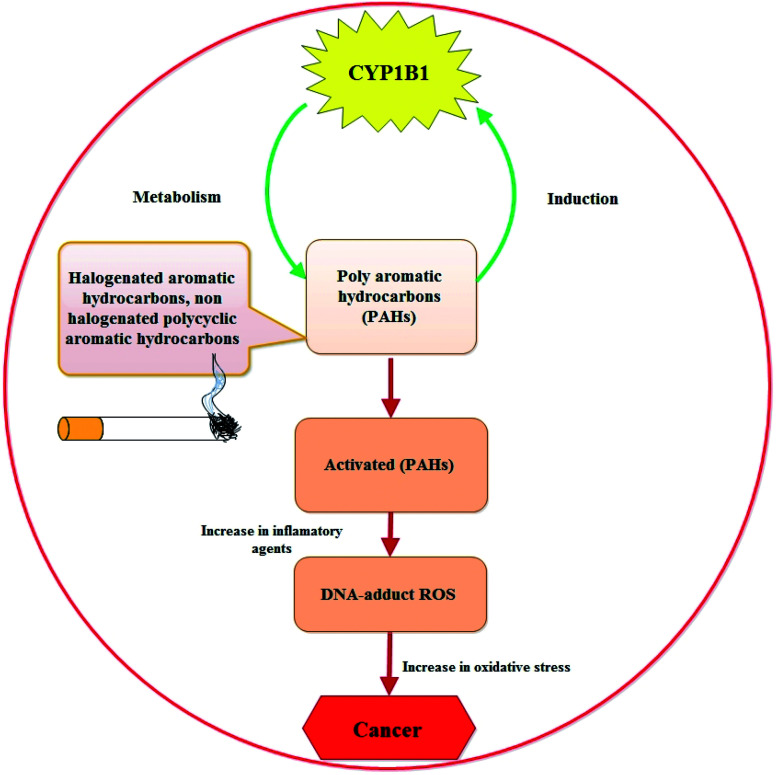
Interplay between CYP1B1 and the development of cancer.

The fact that CYP1B1 is a universal tumor marker states back to 1997, when Professor Burke and research team at Aberdeen University, for the first time, evaluated the presence of CYP1B1 in the cancer cells from different organs (bladder, brain, breast, colon, connective tissue, esophagus, kidney, lung, lymph node, ovary, skin, stomach, testis and uterus) by using standard pathology immunohistochemical techniques and by biochemical western blotting.^16^ After this discovery, different research groups performed similar studies confirming this evidence and regarded CYP1B1 as a biomarker of the neoplastic phenotype and “a shared-tumour associated antigen expressed in almost all human malignancies tested so far” (*i.e.* a universal cancer marker capable of being detected using antibody technology) by staff researchers at the Dana-Farber Cancer Institute (Boston, USA).^45^ Till date, a lot of research work has been done that show higher expression of CYP1B1 in cancer cells of different organs, marking CYP1B1 as a potential contributor to the disease (schematically represented in Fig. 2 describing how CYP1B1 is involved in the etiology of the disease).

Despite the association between CYP1B1 and cancer, the detailed molecular mechanism is still unknown. However, previous reports do indicate that the mRNA expression level of Herc5, an E3 ligase for ISGylation, is promoted by CYP1B1 suppression using specific small interfering RNA, and that ISGylation may be involved in ubiquitination related to β-catenin degradation. These information were supported by the findings of Park *et al.*, who suggested that CYP1B1 may activate Wnt/β-catenin signaling through stabilization of β-catenin protein from Herc5-mediated ISGylation for proteosomal degradation.^46^

Shawn and coworkers stated that bone marrow stromal cells metabolize the polycyclic aromatic hydrocarbon 7,12-dimethylbenz[*a*]anthracene (DMBA) to compounds like 3,4-dihydrodiol, which act as the precursor to the most mutagenic DMBA metabolite. These cells were found to express higher levels of CYP1B1, as well as producing a DMBA metabolite profile that was consistent with CYP1B1 activity. Therefore, it was postulated that bone marrow toxicity may be mediated by CYP1B1-dependent DMBA metabolism, which is regulated by factors other than the aryl hydrocarbon receptor (AhR).^47^

The same research group in the following years, in another study showed that CYP1B1 is responsible for the formation of DMBA dihydrodiol epoxides in the bone marrow. Their results also confirmed the importance of DMBA dihydrodiol epoxide production at the site of cancer development, whereas tissue specific constitutive expression of CYP1B1 may contribute to cancer susceptibility in the human population.^48^ Simon *et al.*, evaluated the human lung expression of CYP1B1 in tumor and non-tumor tissues of 16 individuals through qualitative reverse transcription-polymerase chain reaction and western immunoblots. They concluded that CYP1B1 is commonly expressed in human lung and it may be an important phase I enzyme with respect to human lung carcinogen metabolism.^49^ Marta and research group claimed to be the first who studied the frequency of CYP1B1 polymorphisms in pancreatic cancer as well as the relationship between CYP1B1 variants and mutations in ras genes (K-, H- or N-*ras*) in human neoplasm. They evaluated the m1 allele (Val to Leu at codon 432) and the m2 allele (Asn to Ser at codon 453) CYP1B1 polymorphisms in 129 incident cases of pancreatic ductal adenocarcinoma (PDA). Their findings described that in PDA, polymorphisms in CYP1B1 might be related with K-ras activation pathways.^50^ Another research group examined CYP1B1 polymorphisms in 60 castration-resistant prostate cancer (CRPC) patients treated with docetaxel. They hypothesized that CYP1B1-4326C > G (432LeuVal) polymorphism maybe the possible predictive marker of response and clinical outcome to docetaxel in CRPC patients.^51^ Similarly, Balmukhanov and colleagues studied the associations of polymorphisms in rs1056836 site of *CYP*1*B*1 in 181 breast cancer (BC) patients from two main ethnic groups of Kazakhstan Republic (Kazakhs and Russians). As compared to the controls, the patients from Russian ethnic group exhibited differences in genotype distributions in *CYP*1*B*1 rs1056836 site.^52^ In the same year Majid and research group also conducted a similar study in Iran aiming to investigate *CYP1B1 L432V* polymorphism by selecting 65 lung cancer patients and 80 healthy controls.^53^ Mitsui *et al.*, determined the expression of CYP1B1 in renal cell carcinoma (RCC) cell lines, tissue microarrays of 96 RCC and 25 normal tissues. They also presented the same phenomenon confirming higher CYP1B1 expression in RCC cell lines compared to normal kidney tissue which was found to be linked with tumor grade and stage.^54^ In 2016, researchers from South Korea, explored the same phenomenon and studied the behavior of CYP1B1 in cells expressing higher level of CYP1B1, induced by either 7,12-dimethylbenz[*a*] anthracene (DMBA) or an overexpression vector. They found that CYP1B1 enhances cell proliferation and metastasis by inducing EMT and Wnt/β-catenin signaling *via* Sp1 induction.^55^

## CYP1B1 and hypertension

In recent years, multiple lines of evidence from both humans and mice show a significant role for CYP1B1 in the development of hypertension and associated pathophysiological changes including activation of nicotinamide adenine dinucleotide phosphate oxidase and generation of reactive oxygen species (ROS), inflammation, and endothelial dysfunction.^56^

**Fig. 3:**
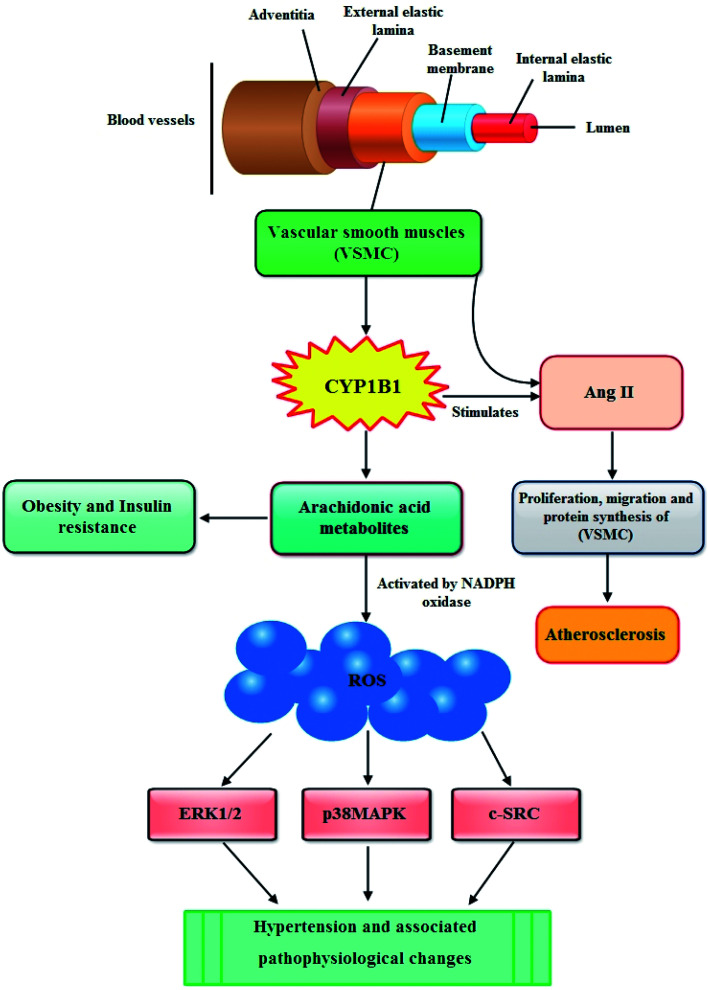
Possible mechanism through which CYP1B1 contributes in the development of cardiovascular diseases.

In blood vessels CYP1B1 is expressed in vascular smooth muscle cells (VSMCs) and the migration, proliferation, and hypertrophy of these VSMCs caused by angiotensin II (Ang II) are mediated by CYP1B1 dependent production of ROS. The enhanced production of ROS causes endothelial dysfunction thus leading to atherosclerosis (Fig. 3).^57^ Moreover, it has also been reported that CYP1B1 contributes in the development of pulmonary arterial hypertension (PAH), a progressive disease of the pulmonary vasculature resulting in right heart failure and death. CYP1B1 is expressed in the lung where it catalyzes the conversion of the 4-hydroxylation of estrogens predominantly to 4-hydroxyestrogens. It also metabolizes estrogen *via* 16-hydroxylation, resulting in formation of the potent mitogen 16 hydroxyestrogen.^58^ Now consistent with these information, Brett *et al.*, (2010) investigated the role of CYP1B1 in the development and maintenance of angiotensin II – induced hypertension by infusing angiotensin II into rats for 13 days. They described that the increase in blood pressure and associated pathophysiological changes were minimized by using CYP1B1 inhibitor 2,3′,4,5′-tetramethoxystilbene, and found markedly reduced in *Cyp1b1*^−/−^ mice. Based on their findings, they demonstrated that CYP1B1 contributes in the development of hypertension most likely by increased generation of ROS, ERK1/2, and p38 MAPK activity; vascular hypertrophy; endothelial dysfunction; and increased vascular reactivity.^59^ The same research group further elaborated this phenomenon and evaluated the role of CYP1B1 in the gender difference in response to Ang II-induced hypertension in wild type *Cyp1b1*^+/+^ and *Cyp1b1*^−/−^ female mice. Their results outlined that in female mice, Cyp1b1 plays a critical role in maintaining the reduced hypertensive effect of Ang II and associated pathophysiological changes, most likely through generation of 2-MeE2 and consequently reduced oxidative stress and activity of signaling molecules, extracellular signal-regulated kinase (ERK1/2), p38 mitogen-activated protein kinase (MAPK), c-Src, and Akt.^60^ Similarly, Kevin and colleagues highlighted the influence of CYP1B1 overexpression and activity associated with the development of pulmonary arterial hypertension (PAH) through possible attribution of pathogenic estrogen metabolism. They stated that enhanced pulmonary CYP1B1 expression was found in hypoxic PAH, hypoxic + SU5416 PAH, and human PAH as well as within the pulmonary vascular wall. However, attenuated hypoxic PAH was observed in *Cyp1b1*^−/−^ mice, whereas the mice administered with potent CYP1B1 inhibitor 2,3′,4,5′-tetramethoxystilbene (TMS) showed significantly attenuated hypoxic PAH, hypoxic + SU5416 PAH. Also, TMS prohibited estrogen-induced proliferation in PAH-pulmonary artery smooth muscle cells. The mitogenic effect of estrogen metabolite 16α-hydroxyestrone was significantly manifested in PAH-pulmonary artery smooth muscle cells and was found to have higher concentration in PAH, consistent with CYP1B1 overexpression and activity. Overall, the findings of this study let them make a conclusion that increased CYP1B1-mediated estrogen metabolism promotes the development of PAH, likely *via* the formation of mitogens, including 16α-hydroxyestrone.^61^ In the following year, Yvonne and research group assessed the role of CYP1B1 in the development of PAH induced by an anorectic drug called dexfenfluramine (Dfen) in female mice. This drug is believed to mediate PAH *via* a serotonergic mechanism and it has been shown that serotonin up-regulate expression of CYP1B1 in human pulmonary artery smooth muscle cells (PASMCs). The mice dosed with Dfen showed increased whole lung expression of CYP1B1 and Dfen-induced PAH was abducted in *Cyp1b1*^−/−^ mice. Dfen up-regulated expression of CYP1B1 in PASMCs from PAH patients (PAH-PASMCs) and Dfen-mediated proliferation of PAH-PASMCs was abducted by pharmacological inhibition of CYP1B1. To summarize their results, it can be concluded that CYP1B1 is critical in the development of Dfen-induced PAH and therefore can be a novel therapeutic target for PAH.^62^ Chi and coworkers last year investigated the contribution of CYP1B1 to the development of atherosclerosis and hypertension and associated pathogenesis in 8 week-old male apolipoprotein E-deficient (*ApoE*^−/−^/*Cyp1b1*^+/+^), and ApoE- and CYP1B1d deficient (*ApoE*^−/−^/*Cyp1b1*^−/−^) mice fed a normal or atherogenic diet for 12 weeks. Concluding their results they suggested that aortic lesions, hypertension, and associated pathogenesis in (*ApoE*^−/−^/*Cyp1b1*^+/+^) mice on an atherogenic diet are supposed to be dependent on CYP1B1-induced oxidative stress and enhanced plasma lipid levels independent of blood pressure and absorption of lipids.^63^

## Role of CYP1B1 in obesity

Obesity, a major global problem caused by excess fat accumulation in adipose tissues, is the leading nutritional disorder in developed as well as developing countries. Besides, its contribution in different pathological conditions like osteoarthritis, gallstones, obstructive sleep apnea, hypoventilation syndrome, and infertility, obesity is considered as an important independent risk factor for metabolic diseases including cardiovascular diseases, type 2 diabetes and certain cancers.^64^ Previous studies have shown that adipose tissue in obesity is in a heightened state of inflammation as highlighted by the increased infiltration of immune cells (macrophages and T cells) producing majority of the inflammatory cytokines.^65^ In this context, Da Young and coworkers conducted a detailed study to track macrophage migration to adipose tissues by labeling monocytes with fluorescent PKH26 dye and injecting into recipient mice. The monocytes appeared as adipose, liver, and splenic macrophages and reached maximum in 1 to 2 days. They determined that after injecting, CCR2 KO monocytes into wild-type (WT) recipients, or WT monocytes to MCP-1 KO recipients, adipose tissue macrophage (ATM) accumulation was found to be reduced by ∼40%, whereas hepatic macrophage content decreased by ∼80%. Nevertheless, by using WT donor cells, ATM accumulation was found to be several-fold greater in obese recipient mice as compared with non-obese, irrespective of the source of donor monocytes. After their appearance in adipose tissue, these ATMs tended to be polarized from the non-inflammatory (M2) to the inflammatory (M1) like state in obesity. These outcomes concluded that the CCR2/MCP-1 system is a controlling factor to monocyte migration into adipose tissue responsible for the appearance of recruited macrophages in the hepatic tissues. Although monocytes from obese mice were not programmed to become inflammatory ATMs however, the increased proinflammatory ATM recruitment in obesity was in response to tissue signals.^66^ Similar, findings have also been reported by Bonnie and Alyssa. They presented a detailed review on the infiltration and recruitment of immune cells (macrophages and T cells) to adipose tissues that are responsible for the production of the majority of inflammatory cytokines which are thought to be involved in pathophysiological conditions related to obesity such as insulin resistance, dyslipidemia, diabetes and cardiovascular diseases.^67^

It is evident from the preceding discussion that obesity is a serious health problem that plays a key role in contributing other life threatening pathologies, thus alarming the scientists for searching the possible ways to overcome its incidence and prevalence. In this regard, biomarkers have been identified that may provide information on the pathological condition of obesity.^68^

Although, till date scarce evidences are available to highlight the role of CYP1B1 in obesity, still there are some reports stating that CYP1B1 is involved in the metabolism of endogenous compounds such steroid hormones and lipids that regulate the metabolism, accumulation and distribution of adipose tissues.^69^ In particular, CYP1B1 metabolizes arachidonic acid, the primary source of fatty acids constituting cell membrane glycerophospholipids, which finally lead to obesity and insulin resistance (Fig. 3).^70^ Available literature also shows that the expression of CYP1B1 increases by adipogenic stimulation in similar ways as transcription factor peroxisome proliferator activated receptor gamma (PPARγ) which is not only vital regulator for adipogenesis but is also required for maintenance of the differentiated state.^19,71^ These findings were further elucidated by other researchers who presented a detailed background of the role of CYP1B1 in fatty acid metabolism both *in vivo* as well as *in vitro*. In first instance, the *in vivo* study was performed by selecting adult C57BL/6J wild-type and CYP1B1 null mice which were fed with high fat diets (HFD) for 6 weeks. They determined that by comparing with wild type counterparts, CYP1B1 null mice attenuated HFD-induced obesity and improved glucose tolerance. Also, the reduction in both body weight gain as well as white adipose tissue in CYP1B1 null mice showed coordinate decreases in fatty acid synthesis (PPARγ, CD36, FAS, SCD-1) and increases in fatty acid oxidation (UCP-2, CPT-1a). Similarly, reduced hepatocyte TG contents were consistent with hepatic Oil-Red-O staining in the CYP1B1 null mice, and also a nutrient sensor for energy homeostasis (AMPK), was found to be activated in both fat pad and liver of these mice. Nevertheless, *in vitro* system, CYP1B1 knock down in C3H10T1/2 cells didn't exhibit prohibitory effects on adipogenesis induced by adipogenic agents IDM (Insulin, Dexamethasone, Methylisobutylxanthine). Overall, these results postulated a complex regulation network between CYP1B1 and energy homeostasis.^72^

In the same year another research group from University of Wisconsin, Madison also presented similar findings. They studied the expression of CYP1B1 in mouse and stated that higher expression during adipogenesis proposes a key role of CYP1B1 metabolism in fatty acid homeostasis. Two types of mice *i.e.* Wild-type C57BL/6j (WT) and CYP1B1-deficient (CYP1B1-ko) mice were fed with either low or high fat diets (LFD and HFD, respectively). They described that CYP1B1-ko resulted in attenuating HFD-induced obesity, enhanced glucose tolerance and prohibited liver steatosis. Also, suppression of lipid droplets in sinusoidal hepatocytes, associated with improved glycogen granules, was a consistent feature of CYP1B1-ko mice. Whats more, Cyp1b1 deletion was found to be responsible for the alteration of the *in vivo* expression of 560 liver genes, such as suppression of PPARγ, stearoyl CoA desaturase 1 (Scd1) and many genes stimulated by PPARα, each consistent with this switch in energy storage mechanism.^20^ Another research group from this institute found that CYP1B1 deletion and retinol deficiency coordinately suppress mouse liver lipogenic genes and hepcidin expression during post-natal development. These neonatal change in CYP1B1 deficient mice were lined to attenuated adult obesity and liver inflammation.^73^ Last year, Falero-Perez and colleagues evaluated the impact of CYP1B1 expression on LSEC (liver sinusoidal endothelial cells) properties. Concluding their findings, it was stated that CYP1B1 expression had a significant impact on LSEC angiogenic and inflammatory functions.^31^ Most recently, the connection between HFD and CYP1B1 modulation in learning and memory deficits was described by Zhuanhong and research group in female C57BL/6J mice. The outcomes of their study concluded that Cyp1b1 deficiency safeguarded mice from HFD induced cognitive impairment. Sustained BDNF (brain derived neurotrophic factor) expression, reduced β-amyloid accumulation and brain oxidative stress, and Nrf2 deactivation were proposed to be the key events in mice redox system through Cyp1b1-diet interaction.^74^ The same group in the same year further stated that knockdown of CYP1B1 suppresses the behavior of the extravillous trophoblast (EVT) cell line HTR-8/SVneo under hyperglycemic condition. Their findings concluded that CYP1B1 is highly expressed in placentas from women with gestational diabetes mellitus. The blockage of CYP1B1 inhibits EVT activities induced by hyperglycemia *in vitro*, including proliferation, migration, and invasion, whereas the exogenous expression of CYP1B1 exhibits the opposite effects.^75^

## Influence of nutrition on CYP1B1

It is a well-known fact that nutrition confers protection against numerous types of chronic diseases such as obesity, diabetes, cardiovascular diseases, osteoporosis and bone fractures, cancer and dental diseases.^76^ In the last decade various nutrients have been recognized to aid in the control and prevention of these diseases by affecting the activities of CYP1B1 (Table 1). Biotin, also known as vitamin H, is one of the B-complex vitamins and has many health implications against diabetes, brittle nails, hair loss, skin rash and mild depression. It acts as a coenzyme for several enzymes *i.e.* acetyl-CoA carboxylase, pyruvate carboxylase, propionyl-CoA carboxylase, and 3-methylcrotonyl-CoA carboxylase, which catalyze important steps in the metabolism of glucose, amino acids, and fatty acids.^77^ Most importantly, biotin affects the expression of a large number of mammalian genes such as CYP1B1, and studies have shown that biotin supplementation increases the abundance of mRNA encoding CYP1B1 in human lymphocytes. In the year (2004), Rocio and coworkers evaluated the signaling pathways that enhance CYP1B1 expression in biotin-supplemented human T (Jurkat) cells. They also examined the link of *CYP1B1* activation with enhanced incidence of single-stranded DNA breaks by culturing the cells in media provided with or without biotin supplements. In their results, they stated that the transcriptional activity of a CYP1B1 reporter gene construct was 24% higher in biotin-supplemented compared with biotin-deficient cells. Similarly, the abundance of CYP1B1 mRNA was 72% greater in biotin-supplemented than in biotin-deficient cells. As determined by electrophoretic mobility shift assays, Sp1 sites in the regulatory region of the *CYP1B1* gene was postulated to play vital roles in transcriptional activation by biotin. The loads of CYP1B1 protein and activity of CYP1B1 were 124 and 35% greater, respectively, in biotin-supplemented compared with biotin-deficient cells. This enhanced expression of CYP1B1 in biotin-supplemented cells was supposed to be linked with an increase in the occurrence of single-stranded DNA breaks compared with biotin-deficient cells. As there were no strand breaks observed when synthetic inhibitors of CYP1B1 were used, it was proposed that the effects of biotin were specific for CYP1B1.^78^ Another important nutrient “resveratrol” a naturally occurring polyphenolic compound found in grapes, peanuts, berries, and a number of plants used in traditional Asian medicine exhibits several beneficial characteristics related to human health, including cardioprotective activity and inhibitory activity toward the ageing process. Furthermore, resveratrol has been reported to demonstrate chemopreventive activity against the development of different types of cancer, at all three stages of carcinogenesis (initiation, promotion, and progression).^79,80^ Several studies also reported that resveratrol inhibits the expression of CYP1B1 in cancer cell lines of different tissue origin thus confirming the cancer chemopreventive activity of this compound.^81^ In 2009, Sudheer *et al.*, presented the mechanism of resveratrol's inhibitory activity toward CYP1B1 induction. They investigated the effect of resveratrol on the recruitment of the AHRC CYP1B1 genes in their natural chromosomal configurations *in vivo*, and thus confirmed that resveratrol inhibits dioxin induction of the CYP1B1 genes. By using the *in vivo* ChIP assay, they described that resveratrol on its own does not induce binding of AHR/ARNT to the enhancer regions of the CYP1A1 or CYP1B1 genes, but does inhibit dioxin's ability to induce this binding, subsequently inhibiting the pol II recruitment to the promoter regions of these genes.^82^

**Table 1:** Association between nutrients and CYP1B1

Nutrients	Mechanism	Action	Conclusion	References
Quercetin (Q), kaempferol (K) and taxifolin (T)	Anti-inflammatory effects of quercetin (Q), kaempferol (K) and taxifolin (T) in J774A	Significantly inhibited the expression of the xenobiotic metabolizing enzyme, cytochrome p450 1B1 (CYP1B1), with potency of inhibition ranked K > Q ≫ T	K exhibited most potent anti-inflammatory effects, and possessed the most potent inhibitory effect on CYP1B1 expression. The anti-inflammatory efficacy of a compound is negatively related to CYP1B1 expression	75
Flaxseed	Effects on different enzymes involved in the oestrogen pathway	Significantly decreased the expression of CYP1B1 in ovaries	Reduced the inflammatory and pro-carcinogenic micro-environment of the ovaries	76
Green tea extracts (GTE)	Effect of GTE supplementation on upregulation of CYP1B1 mRNA in liver cells	GTE supplementation significantly upregulated in CYP1B1 mRNA	Liver samples exhibited potential modulation of its activity in response to GTE diet supplementation, indicating potential for higher toxicant degradation and clearance	77
Indole-3-carbinol (I3C)	Effects on adipogenesis and angiogenesis-associated factors in mature adipocytes	Significantly inhibited triglyceride accumulation in mature adipocytes in association with significantly increased expression of AhR and CYP1B1 proteins	I3C is a potential therapeutic agent for treating obesity and obesity-associated disorders	78
Olive oil	Preventative effects of olive oil on enzo(*a*)pyrene [B(*a*)P]-induced colon carcinogenesis	Significantly altered the expression of drug-metabolizing enzymes (CYP1A1, CYP1B1) in both the colon and liver tissues	Olive oil has a protective effect against environmental toxicant B(*a*)P-induced colon tumors by controlling the levels of CYP1A1, CYP1B1	79
Folic acid	Effect of folic acid (FA) on prevention of cardiac defects during embryo development of zebra fish	Significantly prevented cardiac defects during embryo development by interfering with AhR and Wnt/b-catenin signaling pathways	FA supplementation attenuated the extractable organic matter (EOM)-induced upregulation of AhR and its target genes including Cyp1a1, Cyp1b1, Ahrra, and Ahrrb	80
Piceatannol	Effects of piceatannol against oxidative stress induced cell damage in neuroblastoma cells	CYP1B1 plays a significant role in the conversion of resveratrol to piceatannol (3,5,3′,4′-tetrahydroxystilbene)	Piceatannol stimulates BDNF (brain-derived neurotrophic factor), which is involved in neurite outgrowth and maintenance of function in neuronal cells piceatannol promotes cell survival by sustaining level of sirtuin 1 and seladin-1 mRNA on constant level	81
Apigenin, luteolin, scutellarein, kaempferol and quercetin	Antiproliferative effect of the hydroxylated flavonoids apigenin, luteolin, scutellarein, kaempferol and quercetin in CYP1 combinant enzymes and in the CYP1 expressing cell lines MCF7 and MDA-MB-468, respectively	CYP1 enzymes converted apigenin to luteolin and scutellarein, and kaempferol was metabolized only to quercetin. CYP1B1 metabolized luteolin to 6 hydroxyluteolin	Molecular modeling demonstrated that CYP1B1 favored the A ring orientation of apigenin and luteolin to the heme group. The metabolism of hydroxylated flavonoids by cytochrome P450 CYP1 enzymes, notably CYP1A1 and CYP1B1, can enhance their antiproliferative activity in breast cancer cells	82

Salvestrols is a class of naturally occurring metabolically active substances that is known to act as prodrugs through their activation by CYP1B1. Specifically, CYP1B1 metabolizes salvestrols to produce a metabolite within the cancer cell that induces apoptosis, thus making salvestrols highly targeted dietary rescue mechanism for killing cancer cells.^83^ Interestingly, this defense mechanism of salvestrol shares highly valuable attributes such as; there is no harmful effect as the toxins produced through the metabolism of Salvestrols by CYP1B1 are restricted to the cancer cells solely, and also it involves food and is only based on enzymatic activation and certain cofactors that should be provided by one's daily nutrition.^84^ Similarly, “naringenin” a major bioflavonoid isolated from citrus fruits is believed to exert potential contribution in improving human health. In 2013, Ching and research group examined the inhibitory effect of naringenin on CYP1B1 expression and enzyme activity in MCF-7 cells. They described that by evaluating enzyme inhibition assays, it was found that naringenin inhibited CYP1B1 activity at or above 5 μM. Furthermore, QPCR analysis also showed that 1 μM-naringenin reduced CYP1B1 mRNA expression induced by 7,12-dimethylbenz(*a*)anthracene (DMBA), whereas the suppression was at the transcriptional level. Finally, employing reporter gene assays as well as the electromobility shift assay, they verified that naringenin counteracted DMBA-induced XRE binding at −1675.^85^

Having said that, Arroo *et al.*, presented phytoestrogens as natural prodrugs in cancer prevention. They described that dietary polyphenol *e.g.* isoflavones, coumestans, lignans, flavones and so on have somewhat similar structure with human estrogens which supports the protective roles of these compounds in cancer prevention. Similarly, polymethoxyflavones nobiletin and tangeretin, derived from Citrus peel, better absorbed than polyhydroxy flavonoids, and maintain their biological activity for a longer period of time. The compounds are known to be substrates for the estrogen-converting cytochrome P450 enzymes CYP1A1 and CYP1B1. However, they acknowledged that unfortunately so far there is no satisfactory explanation for the mechanism through which these compounds aid in cancer prevention.^86^ Last year, another research group compared the effects of a western diet, with or without oral benzo[*a*]pyrene (BaP) treatment, on the development of nonalcoholic fatty liver disease (NAFLD) in Cyp1a1(−/−) mice *versus* wild-type mice. Cyp1a1(−/−) mice fed with Western diet and BaP underwent changes in expression of genes involved in bile acid and lipid metabolism, and exhibited enhanced Cyp1b1 mRNA expression, as well as hepatic inflammation.^87^

## Conclusion and future prospective

CYP1B1 belongs to superfamily CYP-450 enzymes which catalyze oxidation of various endogenous and exogenous substrates. It regulates endogenous metabolic pathways, involved in drug metabolism and synthesis of cholesterol, steroids, and other lipids as well as vitamins. The available literature discussed in this article mainly focuses on CYP1B1, its significant contribution in the etiology of different chronic diseases such as primary congenital glaucoma (PCG), cancer, obesity and hypertension in general, and thereafter the role of nutrition (nutrients) on its modulation and expression in particular. These information suggest that CYP1B1 plays a vital role in the progression of these disease and hence can be an effective target of various therapies in their control and treatments. In this context phytochemicals such as dietary flavonoids, being selective inhibitor have shown an outstanding therapeutic potential. Till date a lot of research work has been done evaluating the role of CYP1B1 in the progression of PCG and cancer, however, there is still a large gap in the available literature describing its part in the development of diseases like hypertension, obesity and type 2 diabetes as well. Therefore, based on these observations future work is needed to identify the detail mechanisms of CYP1B1 in the development of these diseases and to search out novel phytochemicals from dietary sources that can aid in their control and treatments.

## Conflicts of interest

There are no conflicts to declare.

## Supplementary Material
